# Lifetime risk of malignancy in polymyalgia rheumatica: a population-based matched cohort study from southern Norway

**DOI:** 10.1093/rheumatology/keag297

**Published:** 2026-06-09

**Authors:** Stig Tengesdal, Øyvind Molberg, Øyvind Holme, Reza Ghiasvand, Jan Tore Gran, Geirmund Myklebust

**Affiliations:** Department of Research, Sorlandet Hospital, Kristiansand, Norway; Department of Rheumatology, Oslo University Hospital, Rikshospitalet, Oslo, Norway; Institute of Clinical Medicine, University of Oslo, Oslo, Norway; Department of Rheumatology, Oslo University Hospital, Rikshospitalet, Oslo, Norway; Institute of Clinical Medicine, University of Oslo, Oslo, Norway; Department of Research, Sorlandet Hospital, Kristiansand, Norway; Institute of Health and Society, University of Oslo, Oslo, Norway; Centre for Biostatistics and Epidemiology, Oslo University Hospital, Oslo, Norway; Department of Research, Cancer Registry of Norway, Norwegian Institute of Public Health, Oslo, Norway; Institute for Cancer Research, Oslo University Hospital, Oslo, Norway; Department of Rheumatology, Oslo University Hospital, Rikshospitalet, Oslo, Norway; Institute of Clinical Medicine, University of Oslo, Oslo, Norway; Department of Research, Sorlandet Hospital, Kristiansand, Norway

**Keywords:** polymyalgia rheumatica, epidemiology, cancer, malignancy

## Abstract

**Objectives:**

Prior studies examining the malignancy risk in polymyalgia rheumatica (PMR) have reported conflicting results, with some indicating an increased risk within 6–12 months after diagnosis. This study assessed malignancy risk over the lifetime and at different follow-up horizons in a population-based inception cohort of PMR patients.

**Methods:**

All incident PMR cases (*n* = 296) in Aust-Agder County, Norway, between 1987 and 1997 were enrolled at diagnosis in a prospective, population-based study. Diagnosis was ascertained clinically by rheumatologists, with all cases fulfilling Bird’s criteria. Each PMR case was matched by age, sex and residency to 15 random comparators (*n* = 4440). Cancer incidence data were obtained from the Cancer Registry of Norway. Participants were followed until first cancer diagnosis, death or 31 December 2024. Malignancy risk was estimated using Cox regression stratified on matched groups.

**Results:**

The mean age at PMR diagnosis was 72 years, 67.6% were female and 95.6% were deceased by 31 December 2024. Median follow-up was 14.1 years in PMR patients and 13.9 years among comparators. Following the index date, 70 PMR patients (23.6%) and 1104 comparators (24.9%) developed malignancy. PMR was not associated with an increased malignancy risk (hazard ratio 0.94; 95% CI: 0.73, 1.21), nor with significant excess risk in separate analyses by cancer type or by time since diagnosis.

**Conclusion:**

In this long-term follow-up of a population-based PMR inception cohort, we found no excess malignancy risk at any time from PMR diagnosis through the end of life. These findings reinforce the notion that PMR is not a cancer-associated disease.

## Graphical Abstract

**Graphical Abstract:**
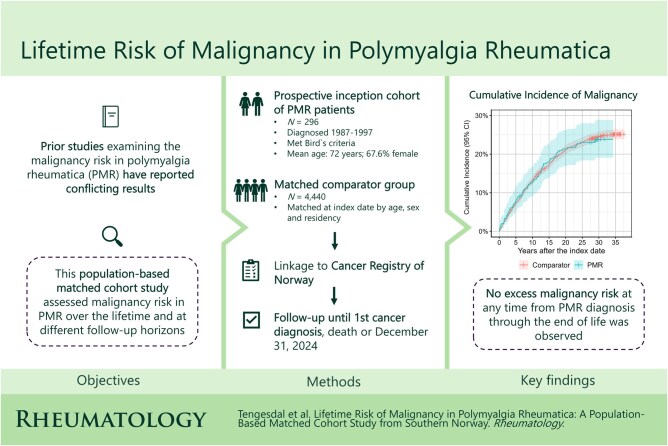

Rheumatology key messages

- Previous studies on PMR and malignancy report conflicting results, including possible short-term increased risk.
- This study found no increased lifetime risk of malignancy following PMR diagnosis.
- Our findings strengthen the evidence against a true association between PMR and malignancy.

## Introduction

Polymyalgia rheumatica (PMR) is an inflammatory rheumatic disease affecting individuals aged 50 years or older, characterized by proximal muscle pain and stiffness, and elevated inflammatory markers [1]. Although these are the hallmark features, some patients may present with atypical symptoms such as malaise, night sweats or unintentional weight loss [2]. The diagnosis of PMR remains challenging due to its non-specific clinical presentation and the absence of a definitive diagnostic test [3]. A variety of conditions, including other inflammatory diseases such as rheumatoid arthritis (RA), infections and malignancies, can mimic the symptoms of PMR [4, 5]. Hence, patients may be initially misdiagnosed with PMR when an alternative underlying condition is responsible for their symptoms.

Over the past decades, associations between cancer and rheumatic diseases have been well established [6]. Certain rheumatic conditions are known to confer an increased risk of malignancy, either in general or for specific cancer types [7]. The relationship between PMR and malignancy has been explored in several studies, with inconsistent findings [8–13]. Notably, two studies reported higher rates of malignancy within the first 6–12 months following PMR diagnosis [10, 11]. Possible diagnostic misclassification, surveillance bias and an underlying susceptibility for cancer have been proposed as potential explanations for this observation [14]. Obviously, incidence of PMR is highest in the age groups where incidence of cancer peaks. It is therefore crucial to clarify whether a true link exists between PMR and malignancy that would necessitate more extensive cancer screening protocols in patients with PMR compared with the general population.

A previous study from Norway including 398 patients with PMR and giant cell arteritis (GCA), conducted as part of a broader clinical and epidemiological project, reported no increased malignancy rates prior to or following disease onset [9]. However, interpretation of the findings from this study was limited by a relatively short follow-up period after diagnosis and few cancer events. In addition, cancer rates were reported for PMR and GCA combined. The present study builds on the previously described population-based inception cohort of PMR patients from Norway [9, 15], aiming to evaluate the lifetime risk of both overall and site-specific malignancies using a matched comparator group. Additionally, we examined how malignancy risk varied at different follow-up times after PMR diagnosis.

## Methods

### Study design and patient population

The present study is a population-based matched cohort study. From 1987 to 1997, 398 cases of PMR and GCA in Aust-Agder County, southern Norway, were included in a population-based cohort study [15, 16]. During the inclusion period, the county had one rheumatology department and no private-practice rheumatologists. All general practitioners and hospital physicians in the county were notified about the study beforehand and asked to refer any new cases of PMR and GCA. The patients were diagnosed by experienced rheumatologists, with PMR cases meeting the diagnostic criteria proposed by Bird *et al.* [17], and GCA was confirmed by a positive temporal artery biopsy. More information on how participants were included has been published previously [16]. The inception cohort were regularly followed until permanent disease remission and drug withdrawal, and data collection continued until 1997 [16].

The list of participants from the original inception cohort was kept by the hospital of southern Norway for quality assurance. The dataset included personal identification numbers, sex, diagnosis (PMR and/or GCA), inclusion dates and place of residence at the time of inclusion. Additional clinical details, including disease characteristics and treatment information, could not be retrieved, as most data from the original inclusion period have not been digitized.

**Figure 1:**
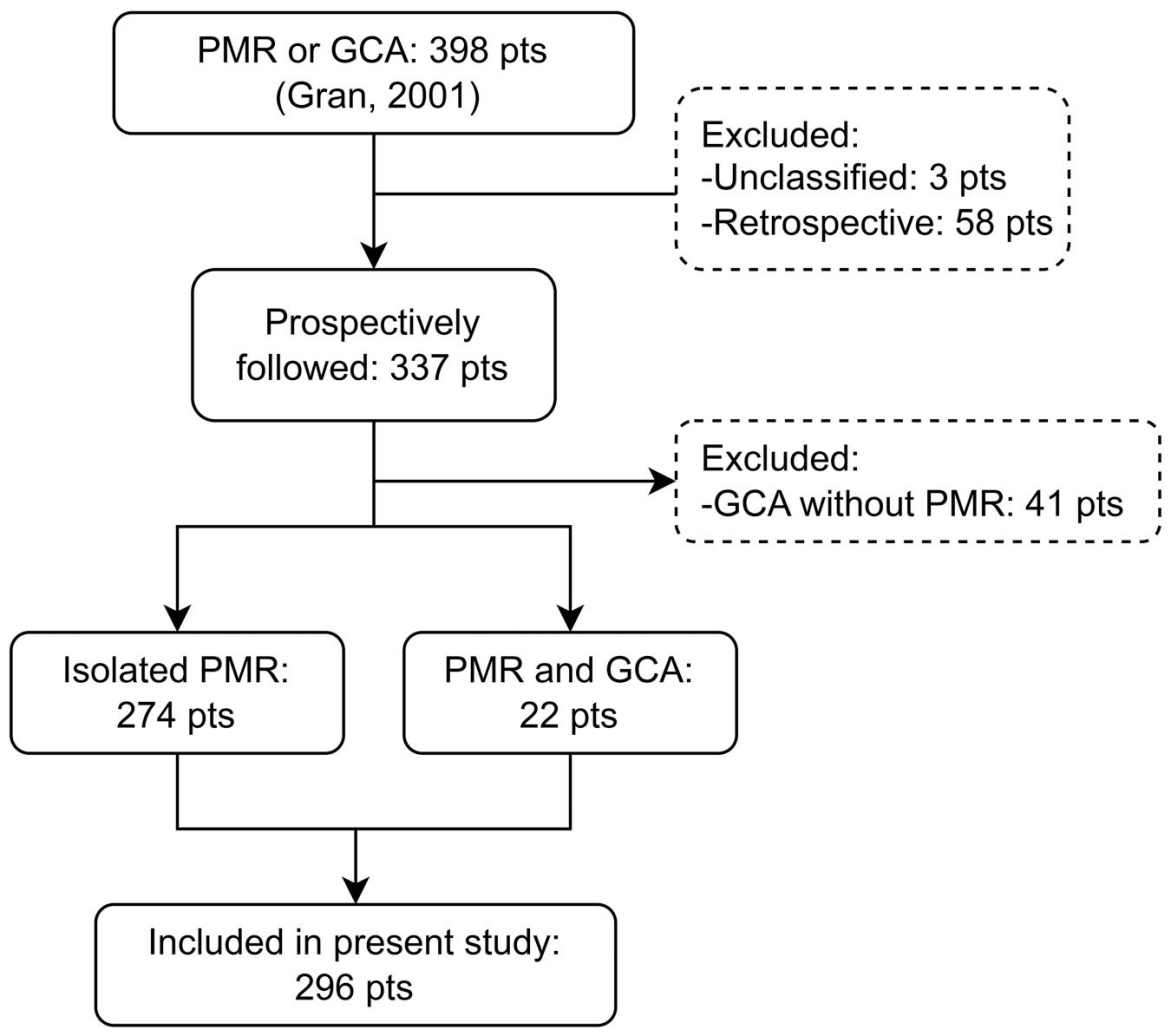
Flowchart illustrating relationships between the original project cohort and inception PMR cohort included in the present study. GCA: giant cell arteritis; PMR: polymyalgia rheumatica; pts: patients

In the present study, we identified and included all 296 cases of PMR from the inception cohort (Fig. 1).

### Comparator group

A matched comparator group was established using data from Norway’s National Population Registry (NPR). In Norway, each resident is assigned a unique national identification number, which links individuals to the health and administrative registries and ensures accurate longitudinal tracking.

For each PMR index case, 15 comparators were randomly selected (without replacement) and matched individually at baseline by the following four parameters: (i) month and year of birth, (ii) sex, (iii) vital status at time of study entry (alive when their corresponding index case was enrolled in the study), and (iv) residency in same county at time of study entry, resulting in a total of 4440 comparators.

### Cancer data and definitions

We obtained cancer incidence data through 31 December 2024, from the Cancer Registry of Norway (CRN), which has systematically recorded all new malignant neoplasm diagnoses nationwide since 1952. Reporting to the CRN is mandatory for all healthcare professionals involved in the diagnosis, treatment or monitoring of cancer patients. This comprehensive reporting system has made the CRN one of the oldest and most complete cancer registries in the world. With the exception of basal cell carcinoma, all cancer diagnoses are required to be reported.

From the CRN, we received the date of cancer diagnosis, the corresponding International Statistical Classification of Diseases and Related Health Problems 10th Revision (ICD-10) code, International Classification of Diseases for Oncology, 3rd Edition codes, the site of origin, and the diagnostic method. We excluded precancerous conditions and lesions of unverified or uncertain malignant potential. Cancer diagnoses were grouped based on the affected anatomical systems, following the classification framework of the ICD-10 coding system.

### Follow-up and outcome definition

Participants were followed from the index date, defined as the date of diagnosis for PMR patients and matched date for comparators, until the first occurrence of any of the following: cancer diagnosis, death, emigration or end of study period (31 December 2024). Vital and emigration status and corresponding dates were obtained from the NPR.

### Statistical analysis

Descriptive statistics were used to summarize the characteristics of patients with PMR and their matched comparators. The prevalence of malignancy prior to the index date was compared between groups using 2 × 2 contingency-table analysis, estimating odds ratios (OR) with 95% CI using the exact method.

The primary outcome was the diagnosis of a first malignancy after the index date. Individuals with a history of malignancy prior to the index date were still considered at risk for developing a new primary malignancy and included in the analysis; but only the post-index events were counted. A sensitivity analysis excluding individuals with prior malignancy was conducted. Follow-up time was estimated using the reverse Kaplan–Meier method. Incidence rates (IR) were calculated as the number of first primary cancers occurring after index date divided by the total person-time at risk, expressed per 1000 person-years. The cumulative incidence of the first primary malignancy after the index date was estimated non-parametrically, accounting for the competing risk of death, and presented graphically. Differences in overall and type-specific malignancy risk were estimated using Cox proportional hazard regression stratified by the matched groups, with results expressed as hazard ratios (HR) and 95% CI. The proportional-hazards assumption was assessed using Schoenfeld residuals. To examine how malignancy risk evolved over time, additional analyses were conducted at predefined follow-up horizons from the index date (6 months, 1, 2, 5, 10 and ≥20 years). All analyses were adjusted for sex and birth year by design.

Statistical analyses were conducted using Stata version 18.0 (StataCorp LLC, College Station, TX, USA). *P-*values <0.05 were considered statistically significant. Graphical plots were created in R studio version 2025.09.2 + 418 (Posit Software, Boston, MA, USA).

### Ethics

This study was approved by the regional ethics committee from the south-east region of Norway (45964) which granted exemption from obtaining informed consent for identifications of patients, and linkage to the NPR and CRN.

## Results

The characteristics and outcome measures for the PMR patients and matched comparators are presented in Table 1. Among the 296 patients with PMR, the mean age at diagnosis was 72.0 years, 200 (67.6%) were female and 22 (7.4%) had coexisting GCA. By the study end on 31 December 2024, 283 PMR patients (95.6%) had deceased. The median time at risk for malignancy after the index date was 14.1 years for patients with PMR and 13.9 years for comparators, yielding a total of 3748 and 55 622 person-years, respectively (Table 1).

**Table 1:** Patient characteristics and malignancy outcomes for patients with PMR and the matched comparators.

Cohort characteristic	**PMR patients (*n*** **=** **296)**	**Comparators (*n*** **=** **4440)**
Age at PMR diagnosis/matching, mean (s.d.), years	72.0 (0.48)	72.0 (0.13)
Female, *n* (%)	200 (67.6)	3000 (67.6)
Isolated PMR, *n* (%)	274 (92.6)	NA
PMR with GCA, *n* (%)	22 (7.4)	NA
Deceased at study end^a^, *n* (%)	283 (95.6)	4259 (95.9)
**Outcome measures**		
Individuals diagnosed with ≥1 malignancy during follow-up (prevalent and incident combined), *n* (%)	78 (26.4)	1418 (31.9)
Individuals with prior malignancy before the index date, *n* (%)	19 (6.4)	407 (9.2)
Time from prior malignancy to index date, median (IQR), years	5.1 (3.0, 15.5)	6.0 (2.2, 14.3)
Person-years at risk for malignancy^b^	3748	55 622
Follow-up, median (95% CI), years^b^	14.1 (13.0, 15.2)	13.9 (13.5, 14.5)
Individuals diagnosed with ≥1 malignancy after diagnosis/index date, *n* (%)	70 (23.6)	1104 (24.9)

a As of 31 December 2024.

b Counted from index date until first new primary malignancy, death or censoring date. Abbreviations: IQR: interquartile range; NA: not applicable.

### Malignancy prevalence

By the study end of 31 December 2024, 78 (26.4%) PMR patients and 1418 (31.9%) of the comparators were diagnosed with malignancy (prevalent and incident cancers combined) (Table 1). The overall prevalence of malignancies prior to the index date did not differ between PMR patients and comparators (OR 0.68; 95% CI: 0.42, 1.09) (Table 2). No differences were observed for any organ-specific cancers (Table 2); however, ORs could not be estimated for several cancer types due to zero events among the PMR patients.

**Table 2:** Prevalence of organ-specific malignancies in PMR patients and matched comparators prior to the index date.

Malignancy group (ICD-10 code)	Individuals with malignancy present before the index date		OR (95% CI)^a^
	PMR patients, *n* (%)	Matched comparators, *n* (%)	
Any malignancy	19 (6.42)	407 (9.17)	0.68 (0.42, 1.09)
Lip, oral cavity and pharynx (C00–C14)	0 (0)	12 (0.27)	—
Digestive organs (C15–C26)	2 (0.68)	71 (1.60)	0.42 (0.05, 1.58)
Respiratory organs (C30–C39)	0 (0)	8 (0.18)	—
Skin melanoma (C43)	2 (0.68)	34 (0.77)	0.88 (0.10, 3.47)
Other skin neoplasms (C44)	2 (0.68)	29 (0.65)	1.03 (0.12, 4.12)
Bone and soft tissue (C40–41, C45–49)	0 (0)	3 (0.07)	—
Breast (C50)^b^	5 (2.50)	78 (2.60)	0.96 (0.30, 2.38)
Female genital organs (C51–C58)^b^	1 (0.50)	67 (2.23)	0.22 (0.01, 1.28)
Male genital organs (C60–C63)^b^	4 (4.17)	39 (2.71)	1.56 (0.40, 4.47)
Urinary tract (C64–C68)	1 (0.34)	33 (0.74)	0.45 (0.01, 2.72)
Eye and CNS (C69–C72)	0 (0)	4 (0.09)	—
Endocrine glands (C73–C75)	2 (0.68)	13 (0.29)	2.32 (0.25, 10.30)
Lymphoid and hematopoietic (C81–C96)	0 (0)	14 (0.32)	—
Other/unspecified	0 (0)	2 (0.05)	—

a Odds ratio with exact CIs were calculated using 2 × 2 contingency tables.

b Opposite sex excluded from respective analysis. Abbreviations: ICD-10: International Classification of Diseases, 10th Revision; OR: odds ratio; PMR: polymyalgia rheumatica.

### Malignancy incidence

**Figure 2:**
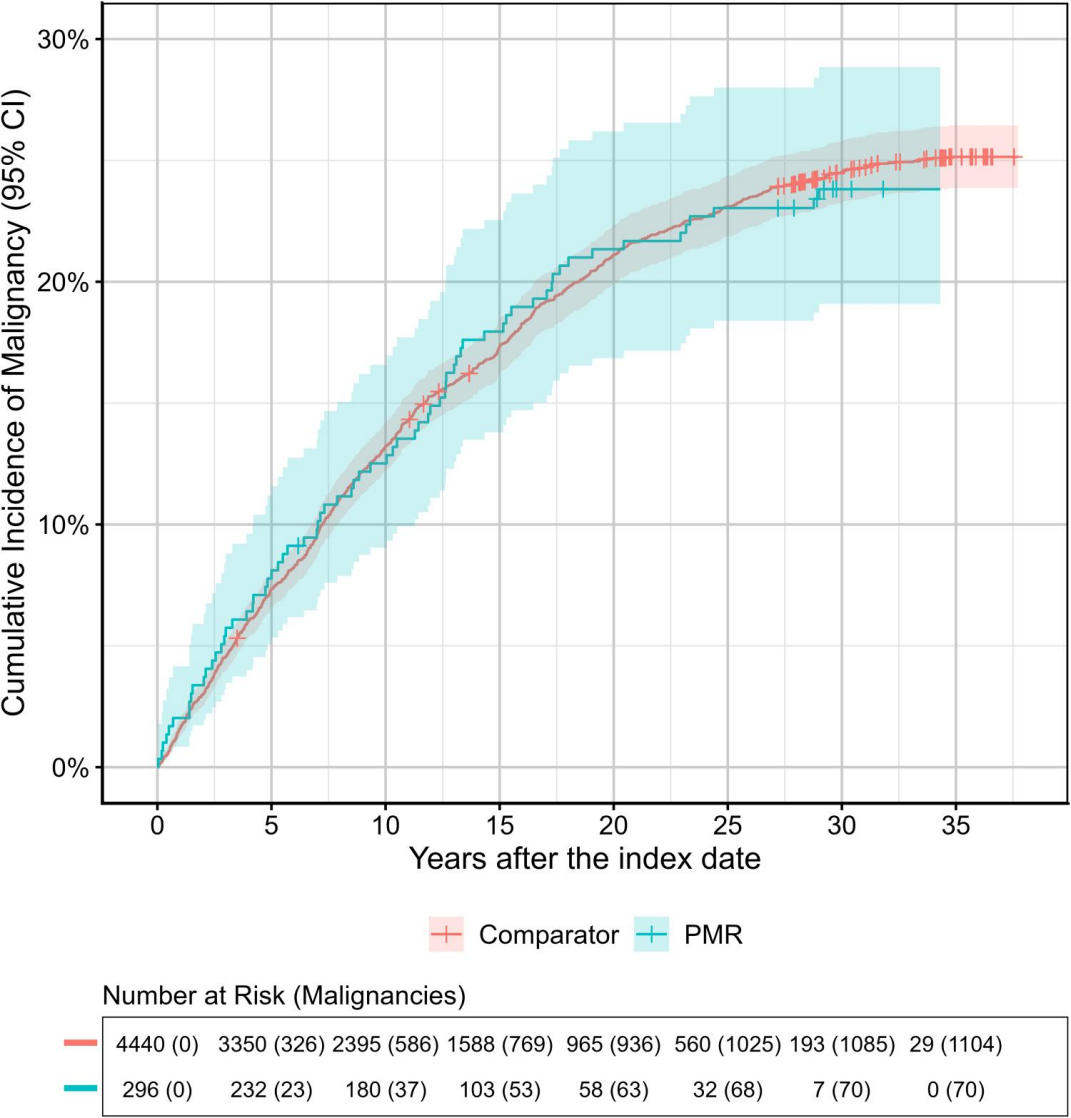
Cumulative incidence of malignancy in PMR patients and matched comparators after the index date, with death as competing risk. Censor marks indicate emigration or being alive without malignancy at study end. PMR, polymyalgia rheumatica

Following the index date, 70 (23.6%) PMR patients and 1104 (24.9%) of the comparators developed malignancy (Fig. 2), with corresponding IRs of 18.7 and 19.8 per 1000 person-years, respectively (Table 3). Being diagnosed with PMR was not associated with an overall increased risk of malignancy compared with comparators; HR 0.94 (95% CI: 0.73, 1.21) (Table 3). These findings remained consistent in a sensitivity analysis excluding individuals with a prior history of malignancy: HR 0.87 (95% CI: 0.66, 1.15). In the sex-stratified analyses, HR for males was 0.87 (95% CI: 0.59, 1.29) and for females 1.00 (95% CI: 0.72, 1.39). Age-group stratified analyses showed no significant differences in malignancy risk (Supplementary Table S1). For PMR patients with coexisting GCA, the HR was 0.80 (95% CI: 0.31, 2.03). There were no significant increases in HRs for any type-specific malignancies (Table 3). Malignancy risk at predefined follow-up horizons after the index date was not significantly different between PMR patients and comparators (Table 4). Details of cancers diagnosed in the year before and after PMR diagnosis are presented in Supplementary Table S2.

**Table 3:** Incidence of organ-specific malignancies in PMR patients and matched comparators following the index date.

Malignancy group (ICD-10)	PMR patients (*n* = 296)		Matched comparators (*n* = 4440)		HR^b^ (95% CI)
	*n*	Incidence rate^a^	*n*	Incidence rate^a^	
Any malignancy	70	18.7	1104	19.8	0.94 (0.73, 1.21)
Lip, oral cavity and pharynx (C00–C14)	0	0	8	0.14	NE
Digestive organs (C15–C26)	10	2.67	282	5.07	0.55 (0.29, 1.03)
Respiratory organs (C30–C39)	7	1.87	121	2.18	0.80 (0.37, 1.74)
Skin melanoma (C43)	6	1.60	41	0.74	2.19 (0.89, 5.39)
Other skin neoplasms (C44)	12	3.20	150	2.70	1.09 (0.55, 2.13)
Bone and soft tissue (C40–41, C45–49)	0	0	10	0.18	NE
Breast (C50)^c^	5	1.96	88	2.19	1.05 (0.42, 2.64)
Female genital organs (C51–C58)^c^	2	0.78	86	2.14	0.37 (0.09, 1.50)
Male genital organs (C60–C63)^c^	11	9.22	134	8.69	1.10 (0.58, 2.08)
Urinary tract (C64–C68)	5	1.33	60	1.08	1.26 (0.43, 3.70)
Eye and CNS (C69–C72)	1	0.27	9	0.16	2.00 (—)
Endocrine glands (C73–C75)	1	0.27	3	0.05	6.08 (—)
Lymphoid and hematopoietic (C81–C96)	7	1.87	65	1.17	1.84 (0.81, 4.15)
Other/unspecified	3	0.80	47	0.85	0.91 (0.26, 3.16)

a Incidence rate calculated as first primary malignancies after index date divided by the total person-time at risk, per 1000 person-years.

b Estimated by Cox regression stratified on matched groups.

c Opposite sex excluded respective analysis. Abbreviations: HR: hazard ratio; ICD-10: International Classification of Diseases, 10th Revision; NE: not estimable due to absence of events in the PMR group; PMR: polymyalgia rheumatica.

**Table 4:** Risk of malignancy after the index date at increasing follow-up times.

	PMR		Matched comparators		**HR** ^b^ (**95% CI)**
	*n*	Incidence rate^a^	*n*	**Incidence rate** ^a^	
0–6 months	4	27.2	32	14.6	1.84 (0.65, 5.20)
12 months	6	20.6	76	17.6	1.18 (0.51, 2.72)
2 years	11	19.4	145	17.3	1.08 (0.59, 2.00)
5 years	23	17.3	341	17.7	0.99 (0.65, 1.52)
10 years	39	16.7	606	18.0	0.91 (0.65, 1.26)
20 years	63	18.5	948	19.1	0.95 (0.73, 1.23)
38 years	70	18.7	1104	19.8	0.94 (0.73, 1.21)

a Incidence rate calculated as first primary malignancies after index date divided by the total person-time at risk, per 1000 person-years.

b Estimated by Cox regression stratified on matched groups. Abbreviations: HR: hazard ratio; n: number of individuals with malignancy. PMR: polymyalgia rheumatica.

## Discussion

In this population-based cohort study with nearly complete lifetime follow-up of 296 prospective patients with PMR, we found no overall increased risk of malignancy compared with 4440 comparators matched by age, sex and residency. Furthermore, no significant increase in the risk of any cancer type was observed, and malignancy risk did not differ significantly at any time point after PMR diagnosis.

Previous studies investigating the risk of malignancy after PMR diagnosis have yielded inconsistent findings. Two prior cohort studies from Norway, including a short-term follow-up on our patient cohort, reported no increased rates of malignancies among PMR individuals [8, 9]. Furthermore, a population-based cohort study from Olmsted County, Minnesota, USA, identified 359 individuals with PMR from medical charts and observed no increased risk of malignancy over a mean follow-up of 11.8 years compared with 357 comparators [12].

In contrast, three other studies reported increased malignancy rates among patients with PMR [10, 11, 13]. A hospital-based case–control study from Italy, including 100 PMR patients identified from medical chart review, reported a marked increased OR of 5.1 of developing cancer compared with osteoarthritis controls [13]. Additionally, in a large Swedish registry study of 35 918 patients with PMR and/or GCA identified from hospital discharge codes, a marginally elevated cancer risk was observed, with the highest incidence occurring within the first year after hospitalization [10]. Likewise, a United Kingdom cohort study using the General Practice Research Database identified 2877 patients with PMR and demonstrated a significant excess of malignancies during the first 6 months following diagnosis compared with the matched controls [11]. Patients in this study were identified by medical codes and required to have at least two corticosteroid prescriptions as a proxy for diagnosis. A key strength of this study was its use of a large primary care population, which reduced selection bias and improves generalizability. A subsequent meta-analysis pooling four of the aforementioned studies [8, 9, 11, 12], together with two additional cohorts of patients with GCA, demonstrated a modest increase in overall risk among patients with PMR/GCA [18]. This excess risk was largely confined to the first 6–12 months after diagnosis (risk ratio 2.16). However, this association was no longer statistically significant in a sensitivity analysis excluding a study with selection bias, precluding any definite conclusions.

Regarding the association of PMR with certain type of cancers, a few case–control studies have reported higher rates of certain haematological malignancies [19–21], whereas others found no such association [22]. In prior studies of incident PMR cases, limited evidence indicated that haematological, lymphoid and prostate cancer may be more common among PMR individuals [10, 11, 14]. Although several reports describe the occurrence of PMR near the time of cancer diagnosis, prior reviews have found limited evidence for PMR as a paraneoplastic phenomenon [14, 23]. In our study, although not significant, the rates of haematological and lymphoid malignancies, as well as melanoma, tended to be higher among PMR patients after the index date, despite their lower prevalence prior to the index date in comparison to the comparator group (Table 3). Of the six cancers diagnosed within 12 months of PMR diagnosis, three were prostate cancers (Supplementary Table S1). Since the number of cancers was few, these findings should be interpreted with caution, as they may reflect random variation rather than true underlying differences.

In summary, cohort studies with higher diagnostic accuracy for ascertaining PMR diagnoses, including the present study, have not demonstrated an increased malignancy risk. This contrasts with findings from case–control and large registry-based cohorts, which provide higher statistical power but rely on less precise diagnostic ascertainment for PMR. In our study, cancer incidence was comparable between PMR patients and comparators. Although a slight increase in risk was observed during the first 6 months after PMR diagnosis, the estimate was driven by a small number of events and substantial statistical uncertainty. This apparent early increase in malignancy rates may reflect lead-time bias, where increased diagnostic or surveillance intensity around the time of PMR diagnosis resulted in earlier detection of pre-existing cancers. The absence of an elevated cancer risk beyond this period provides further support for this explanation, as pre-existing cancers in individuals who did not undergo comprehensive health work-up at that time are likely to be detected at a later time point. Methodological limitations of prior studies may also contribute to the reported early excess cancer risk rather than a casual association. Cancer may initially be misclassified as PMR, as cancer can present with PMR mimicking symptoms and lack other tumour-specific signs early, particularly in studies relying on diagnostic codes as case definition of PMR. Finally, hospital-based studies are prone to selection bias, since patients with underlying cancer are more likely to be hospitalized.

Given the comparable overall mortality observed between our patients with PMR and matched comparators during follow-up in a recent publication [24], it is unlikely that the competing risk of death masked higher malignancy rates through earlier mortality and thereby reduced the time at risk for cancer in the PMR cohort.

The main strength of the present study lies in the near complete life-time follow-up of a well-defined inception cohort of individuals with PMR. By design, the cohort is population-based and presumably includes every incident PMR case withing the area during the inclusion period, providing high generalizability of our results. At the time, this was enabled by close collaboration with primary physicians and hospital specialists, as well as short geographical distances within the study region. The population-based design, with comparators drawn as a random sample from the general population, also minimized the risk of selection bias.

Additional strengths include diagnostic validity, as all PMR cases were ascertained by an experienced rheumatologist with clinical follow-up until permanent disease remission or drug withdrawal [16]. Finally, linkage to the CRN, which has maintained high-quality, nationwide registration since 1952, ensured complete data on cancer diagnoses in our cohort. This provides unique and robust estimates on cancer incidence for the PMR cohort during their lifetime.

As noted previously, the main limitation of this study is the limited power to detect modest risk differences in cancers for several subgroup analyses due to few events, including during the first years of follow-up after PMR diagnosis. Consequently, the estimates for these subgroup analyses are uncertain and should be interpreted with caution. Due to the lack of digitized medical records, we did not have access to information on smoking status and other established cancer risk factors. However, given that established cancer risk factors are not associated with an increased risk of PMR, it is unlikely that these factors confounded the observed associations or biased our estimates. A previous study of a subset of our PMR cohort enrolled between 1987 and 1993 reported that 11 of the PMR patients later developed RA. Among these 11, there were three seropositive cases [25]. In the present study, we were not able to identify and exclude these cases as the original clinical data were no longer available. However, the average interval from PMR onset to RA development was 5.2 years [25], indicating low likelihood of misdiagnosis.

In the context of the existing literature, our findings further strengthen the evidence against a true association between PMR and malignancy. In line with the conclusions of the study by Pfeifer *et al.*, our findings suggest that cancer screening for PMR patients could align with established age and sex cancer screening guidelines for the background population [12]. However, since PMR lacks pathognomonic features and a gold standard diagnostic test, distinguishing true PMR from mimicking conditions can be challenging in clinical practice. In atypical presentations, such as those with concomitant fever, absence of prolonged morning stiffness or poor response to glucocorticoids, more comprehensive diagnostic investigations and closer follow-up are warranted [26].

In conclusion, in this population-based matched cohort study, we found no increased lifetime risk of malignancy in patients diagnosed with PMR compared with the matched population comparators. Our findings support the use of established national cancer screening guidelines based on age and sex for patients with PMR.

## Supplementary Material

keag297_Supplementary_Data

## Data Availability

The data underlying this article will be shared on reasonable request to the corresponding author.
