# Supplementary material for: Lifetime risk of malignancy in polymyalgia rheumatica: a population-based matched cohort study from southern Norway
Source: Rheumatology (Oxford). 2026 Jun 9;65(6):keag297. doi: 10.1093/rheumatology/keag297 (PMC13282711; doi:10.1093/rheumatology/keag297)
Supplement: keag297_Supplementary_Data [file keag297_supplementary_data.docx]

**Supplementary file**

**Tengesdal et al. Lifetime Risk of Malignancy in Polymyalgia Rheumatica: A Population-Based Matched Cohort Study from Southern Norway.**

**Supplementary Table S1:** Age-group stratified malignancy risk in PMR patients versus matched comparators

| Age group | PMR patients | | | Matched comparators | | | HR† | 95% CI |
| --- | --- | --- | --- | --- | --- | --- | --- | --- |
|  | N | N of malignancies | Incidence rate* | N | N of malignancies | Incidence rate* |  |  |
| 50-59 | 31 | 10 | 15.7 | 449 | 170 | 17.1 | 0.94 | 0.49, 1.80 |
| 60-69 | 91 | 20 | 13.2 | 1,367 | 426 | 19.0 | 0.67 | 0.42, 1.08 |
| 70-79 | 121 | 25 | 19.9 | 1,833 | 383 | 20.7 | 0.97 | 0.63, 1.50 |
| ≥80 | 53 | 15 | 44.0 | 791 | 125 | 26.0 | 1.68 | 0.96, 2.97 |
| **Abbreviations:** PMR, polymyalgia rheumatica; N, number; HR, hazard ratio; CI, confidence intervals.  * Incidence rate calculated as first primary malignancies after index date divided by the total person-time at risk, per 1,000 person-years.  † Estimated by Cox regression stratified on matched groups. | | | | | | | | |

**Supplementary Table S2:** Malignancy diagnoses within one year before and after PMR diagnosis

| Diagnosis | Malignancy | Metastatic disease | Time from PMR diagnosis to cancer diagnosis (weeks) |
| --- | --- | --- | --- |
| PMR and GCA | Breast | Unknown at time of cancer diagnosis | 6-9 months before |
| PMR | Digestive organs | Regional metastasis | 3-6 months before |
| PMR and GCA | Prostate | No | 0-3 months |
| PMR | Prostate | No | 0-3 months |
| PMR | Respiratory organs | No | 0-3 months |
| PMR | Lymphoid and hematopoietic | Unknown at time of cancer diagnosis | 3-6 months |
| PMR | Prostate | No | 6-9 months |
| PMR | Lymphoid and hematopoietic | Unknown at time of cancer diagnosis | 6-9 months |
| **Abbreviations:** PMR, polymyalgia rheumatica; GCA, giant cell arteritis. | | | |
